# Obesity Among Healthcare Workers in Al Ahsa, Saudi Arabia: Prevalence, Predictors, and Workplace Health Implications

**DOI:** 10.3390/healthcare13050528

**Published:** 2025-02-28

**Authors:** Salwa Hassanein, Alissar Al Khatib, Omayma AlMoosa, Amany Abdrbo

**Affiliations:** 1 Department of Nursing, Faculty of Health Sciences, Almoosa College, Al Mubarraz 31982, Saudi Arabia; s.hassanein@almoosacollege.edu.sa (S.H.); omayma@almoosacollege.edu.sa (O.A.); 2 Department of Community Health Nursing, Faculty of Nursing, Cairo University, Cairo 12613, Egypt; 3 Department of Nursing, Nursing Management and Informatics, Faculty of Health Sciences, Almoosa College, Al Mubarraz 31982, Saudi Arabia; a.abdrbo@almoosacollege.edu.sa

**Keywords:** obesity prevalence, healthcare employees, predictors of obesity, Saudi Arabia

## Abstract

**Background/Objectives:** Obesity is an emerging public health problem in the world, and health professionals are most likely to be exposed to several occupational determinants thereof. These include long working hours, shift work, high job stress, irregular food intake, poor opportunities for healthy eating, and physical inactivity at work. Healthcare workers’ stressful jobs typically lead to poor eating habits and less opportunity for physical exercise, contributing to obesity risk. The primary objective of this study is to determine the prevalence of obesity among healthcare employees and identify unique predictors that contribute to obesity in this population. **Material and Methods**: Data were collected between 2021 and 2023 from 557 participants through anthropometric measurement and a structured questionnaire using a stratified random sampling technique. **Results:** The study found that 18.6% of participants were classified as obese or morbidly obese (12.9% and 5.7%, respectively), while 33.8% were overweight. The strong predictors of obesity included older age (>30 years, AOR = 2.404, *p* < 0.001) and working in nursing services (AOR = 1.999, *p* = 0.003). Furthermore, 34.3% of respondents had no physical activity, 71.5% slept less than 8 h per day, and 58.5% consumed fast food one to two times per week. A significant association of obesity was found with gender (*p* < 0.001), females being at higher risk, and type of department (*p* = 0.002), nursing staff being at higher risk. However, the predictable factors for obesity did not include nationality, family size, hours of TV watching, and fast-food consumption. **Conclusions:** These findings highlight a significant burden of obesity among healthcare employees, underscoring the need for workplace interventions. The strategy to address obesity among this highly vulnerable population should be directed toward enhancing physical activities, improving eating habits, and managing occupational stress, particularly for older workers and nursing staff.

## 1. Introduction

Obesity is a rapidly growing global health concern with serious implications for public health international perspectives.

The World Health Organization [1] defines obesity as a pathological fat accumulation that results from an excessive or abnormal increase of body fat due to compromised health, as viewed mainly in the context of diabetes and cardiovascular diseases. Globally, its contribution to morbidity and mortality has seen a significant shift. Although it affects all populations, health professionals are prone to it due to the nature of their work, which encompasses stress, irregular eating habits, and a sedentary environment [2]. Despite their medical knowledge, healthcare professionals often lead unhealthy lifestyles, making them susceptible to obesity.

By 2022, an extremely alarming percentage of 40.6% of Saudi adults was predicted to be obese [3]. Surprisingly, few studies are available on obesity and its predictors among health professionals. Healthcare workers play a crucial role in patient care; their well-being directly impacts healthcare quality. Poor health among health professionals affects healthcare delivery through enhanced burnout, absenteeism, and overall productivity [4]. The determination of the prevalence and risk factors is vital for the well-informed creation of policies that may encourage healthy lifestyle changes to preserve augmented workforce resilience.

This research gap will be subsequently addressed by the assessment of the prevalence of obesity and the identification of some of its major drivers among Saudi Arabian healthcare workers, thus contributing to the literature. Healthcare workers include doctors, nurses, and allied health professionals, along with all other staff working in the healthcare sector, including administrative staff and support workers. The demographic, behavioral, and health-related aspects assessed herein are highly influential on the lifestyle and work-related issues of health professionals. This assessment will help in formulating policies and workplace wellness programs that can reduce the obesity rate among medical professionals and improve their quality of life.

## 2. Literature Review

This section offers a systematic structure analysis of regional and worldwide obesity trends, the particular dangers that healthcare workers confront, the main factors that predict obesity in this population, and possible workplace solutions to address the problem.

### 2.1. Prevalence of Obesity in the World and Regionally

Obesity is a major global health concern; in 2016, the WHO estimated that over 650 million adults were classified as obese [1]. The Middle East has reported some of the highest rates of obesity across the globe, due in part to rapid urbanization, increasingly sedentary lifestyles, and shifts in dietary patterns [2]. This trend is very well elaborated in Saudi Arabia, where national data show an increase in its obese adult population. This is an alarming rise, considering the established strong association between obesity and chronic conditions such as diabetes, hypertension, and cardiovascular diseases [3]. While extensive research has examined obesity in the general population, less attention has been given to healthcare workers, a group expected to lead by example in promoting healthy lifestyles.

### 2.2. Obesity Among Healthcare Employees: A Hidden Crisis

Healthcare workers are not an exception, even with their medical knowledge of the condition. Studies have pointed out that this group is generally prone to specific occupational hazards that include long working hours, work shift irregularity, and stress, which ultimately result in the adoption of a very unhealthy lifestyle. Schembri, Heinz, and Samuel [5] found that adults in sedentary occupations had a higher obesity risk due to low physical activity and poor diet. This evidence suggests that healthcare workers, regardless of their knowledge of obesity risks, encounter universal barriers to maintaining a healthy weight. Identifying these occupational and lifestyle challenges is necessary in developing interventions to investigate obesity among healthcare professionals.

### 2.3. Predictors of Obesity

#### 2.3.1. Age and Metabolic Change

There are several factors identified as predictors of obesity among health professionals. Age is one of the key determinants, as older workers are more likely to be obese because of reduced physical activity and metabolic changes associated with aging [6].

#### 2.3.2. Gender and Sociocultural Barriers

Gender is also a factor, as some studies have indicated that female health professionals are more likely to develop obesity than their male colleagues, partly because of the sociocultural barriers to regular physical activity [7]. Almutairi et al. [2] reported that 35% of female nurses in Saudi Arabia were obese, compared to 22% of male nurses. Job titles also play an important role. For instance, nurses and other clinical personnel tend to be in a highly vulnerable position to obesity due to the heavy physical and mental demands of their jobs [4]. For this population, the behavioral factors that have been strongly associated with obesity include physical inactivity, poor dietary habits, and lack of adequate sleep. In this respect, Abd El-Ghany et al. [8] found sleeping less than 8 h each night to be an independent predictor of obesity among healthcare workers. Moreover, they identified frequent consumption of fast foods and poor regular exercise as major contributing factors that led to obesity in healthcare professionals throughout Saudi Arabia.

### 2.4. Workplace Interventions

#### 2.4.1. Wellness Programs and On-Site Facilities

Targeted workplace interventions hold the key to addressing obesity among health employees. Indeed, studies have established that workplace wellness programs, ranging from on-site exercise facilities to nutrition education and stress management, can go a long way in reducing obesity and improving overall health [9]. For instance, when access to the gym and healthier meals were provided in healthcare facilities, Al-Qahtani [3] reported improved health indicators among employees. Moreover, work-hour-related and work–life balance policies have been observed to decrease obesity risk factors [4].

#### 2.4.2. Research Gaps and the Need for Context-Specific Studies

Although studies dealing with obesity among health workers are on the rise, some important gaps remain, particularly concerning the Saudi Arabian context. Most of the studies so far have been conducted in Western countries, and findings may not generalize to the Middle East due to cultural and socio-economic differences. Furthermore, there is scant examination of some specific issues pertaining to healthcare employees in Saudi Arabia, including shift work and cultural barriers to physical activity, particularly in females. The current study will attempt to fill these gaps by conducting a comprehensive analysis of the prevalence and predictors of obesity among healthcare employees in a Saudi Arabian healthcare facility.

In conclusion, improving the health and well-being of healthcare workers requires an inquiry of the prevalence, predictors, and workplace interventions for obesity. International studies have identified key risk factors, but more regional research is required to create adapted therapies that address the particular difficulties Saudi Arabian healthcare personnel confront. By thoroughly analyzing obesity among Saudi Arabian healthcare workers, this study seeks to close these research gaps and offer data-driven insights for future research and workplace health policy.

### 2.5. Significance

Obesity remains a very important global health issue due to its nature, which predisposes individuals to acquiring chronic diseases [1]. The sedentary nature of work, even that of medical workers, improper timing of meals, and chronic stress are all contributory factors that lead to obesity [5]. Due to the many reasons, there is a need to determine the prevalence of obesity and associated risk factors in citizens of Saudi Arabia. First, because health professionals are generally considered the backbone of health systems, poor quality of care provided by them has a direct effect on the health of the patients [10]. Poor health in the working population affects healthcare delivery through increased burnout, absenteeism, and lower productivity. Second, the identification of these predictors of obesity may lead to workplace wellness initiatives promoting physical activity, healthy eating, and stress reduction [8]. These may eventually result in a healthier workforce, with a reduction in obesity and its resulting health problems. Third, the findings of the study have implications for policy interventions aimed at system-level factors such as long working hours, shift work, and work stress to improve the health and well-being of health workers [4]. The practice of community health nursing also will be greatly affected by this study. It is, therefore, recommended that, if the incidence and determinants of obesity in this group are known, community health nurses can use this information to assist in the development of focused interventions, advocacy for policy changes, and reduction of risks of obesity-related illnesses such as diabetes and cardiovascular disease. Insights from this study may, therefore, help develop evidence-based tactics aimed at improving healthcare service quality and staff health, further establishing healthcare professionals as healthy lifestyle role models. The current review, therefore, adds to the growing literature on occupational health, with some helpful recommendations on how healthcare facilities can help address obesity among employees for improved worker productivity and resilience.

### 2.6. Objectives and Research Questions

The primary objective of this study is to determine the prevalence of obesity among healthcare workers and identify unique predictors that contribute to obesity in this population. Specifically, the study questions are the following:

1. What is the prevalence of obesity among healthcare employees in the facility?
2. What are the demographic, behavioral, occupational, and health-related factors that contribute to obesity among healthcare professionals?
3. What predictors are associated with obesity among healthcare employees?

## 3. Materials and Methods

### 3.1. Study Design

This study utilized a predictive cross-sectional design to assess obesity prevalence and predictors among healthcare employees in a healthcare-facility-based setting. Cross-sectional studies are suitable for determining prevalence and identifying associations between variables at a specific point in time [11].

### 3.2. Study Setting

The study was conducted in a large healthcare facility in Saudi Arabia, which employs a diverse workforce, including physicians, nurses, administrative staff, and support personnel. This setting ensures a representative sample of healthcare employees across various roles.

### 3.3. Study Population

The target population includes all full-time healthcare employees aged 18 years and above working in the healthcare facility at Al Ahsa. Al Ahsa is a historically important region located in the Eastern Province of Saudi Arabia. It is famous for its excellent farming regions, developed cities, and cultural heritage. Al Ahsa is home to over 1.3 million residents, and as a result, it has experienced significant development, economic expansion, and urbanization. The region has also seen altered eating patterns and lifestyle choices, with its inhabitants promoting overall better health. The region is most suitable to study obesity among healthcare professionals, as it contains many important healthcare resources, including public and private hospitals and specialized clinics. There is high obesity prevalence in Saudi Arabia, and obesity rates are on the rise, with 40.6% [3] of adults classified as obese, and Al Ahsa serves as a representative example of the national trends. This situation provides an opportunity for a detailed analysis of obesity among healthcare employees in both urban and rural areas. Al Ahsa has unique workplace and cultural issues that could lead to obesity among medical personnel. These consist of extended workdays and shift changes, especially at government hospitals and emergency rooms [12].

### 3.4. Sampling

A sample size of 557 participants was recruited using stratified random sampling to ensure representation across different job categories (e.g., clinical, administrative, and support staff). The health workforce has a number of occupational groups, like doctors, nurses, allied healthcare professionals, and administrative staff, which enhance the representativeness of the sample. Stratified variability in risk factors allowed the study to observe how risk factors affect obesity in different subgroups, leading to more precise conclusions. Random selection in each stratum lowers the chance of selection bias and ensures that there are an equal number of participants in each category, which makes comparative analysis easier.

The sample size was calculated using Cochran’s formula, assuming a 50% prevalence of obesity, a 95% confidence level, and a 5% margin of error [13]. Given the observed prevalence of 18.6%, the study was adequately powered to detect this prevalence; however, it may have limited power to identify smaller effect sizes for predictors.

### 3.5. Data Collection and Tools

A paper-based questionnaire served as the primary data collection tool, ensuring a structured and consistent approach. Paper-based surveys were used due to limited digital access for certain staff members. Participants received printed copies with predefined questions, allowing for accessibility in settings. This method enabled respondents to complete the survey at their own pace, with manual collection for data entry and analysis. Data were collected in 2021.

#### 3.5.1. Data Collection Period

Data collection started in 2021, following the approval from the Institutional Review Board (IRB), and ran over a consecutive two-year period. The extended period was particularly chosen to have a whole and representative sample of healthcare personnel from various departments and shifts. A paper-based standard questionnaire was administered for two years to maintain consistency in collecting the data. Data collectors were appropriately trained to collect data in a consistent and precise manner, following guidelines from data collection and preparation best practices.

#### 3.5.2. Anthropometric Measurements

Height and weight were measured using standardized equipment following established protocols. Body mass index (BMI) was calculated using the formula: weight (kg)/height (m^2^). Obesity was defined as a BMI ≥ 30 kg/m^2^ by the World Health Organization guidelines. Data collectors were appropriately trained to collect data on anthropometric measurements in a consistent and precise manner, following guidelines from data collection and preparation best practices.

#### 3.5.3. Survey Questionnaire

A structured questionnaire was administered to collect comprehensive data on various variables. Demographic variables included age, gender, nationality, family size, job role (e.g., physician, nurse, administrative staff, support staff), and working hours. The prevalence of obesity was assessed through BMI calculations using anthropometric measurements. Behavioral factors were evaluated, including exercise habits (frequency, duration, and type of physical activity), barriers to exercise, dietary habits (frequency of fast-food consumption), and sleep patterns (average hours of sleep per night). Additionally, health status was examined through self-reported comorbidities (e.g., diabetes, hypertension, cardiovascular diseases) and family history of chronic illnesses (e.g., obesity, diabetes, cardiovascular diseases). This comprehensive data collection provided a detailed understanding of the factors influencing obesity among healthcare employees. To enhance the validity of our questionnaire, we reviewed the literature extensively to inform its development. Experts in the field assessed the face validity. Face validity is the extent to which a test appears to measure what it is intended to measure, as defined by an expert. The experts went through the questionnaire to make sure that it properly measured the constructs and that the items were relevant and easy to understand. Their feedback was utilized in refining the questionnaire to make it easier to complete and more appropriate for the study. Some items were adopted from Jaoua, Woodman and Withers who studied the predictors of overweight and obesity among employees [14].

### 3.6. Data Analysis

The data were analyzed using Statistical Packages for Social Sciences (SPSS) version 21. Descriptive statistics were presented using numbers and percentages. Analysis of the association between the level of BMI and socio-demographic characteristics of employees was conducted using Chi-square tests. A multivariate regression analysis was undertaken as well, to predict the influence on obesity of the selected socio-demographic characteristics of employees; the odds ratio and 95% confidence interval were also reported. A *p*-value cut-off point of 0.05 at 95% CI was used to determine statistical significance.

## 4. Results

The entire cohort of 557 employees contributed to this study, with ages ranging from 18 to 65 years (mean age: 32.7 ± 7.17 years), with 47.8% under 30 (Table 1).

The sample primarily comprised females (70%) and non-Saudis (68.2%), with over half (53.5%) reporting fewer than five family members. The majority were employed in nursing services, while the remainder were distributed across administration (15.3%), support (15.8%), and medical services (8.1%).

Regarding BMI categorization, 43.3% of participants had a normal weight, 33.8% were classified as overweight, and 18.6% fell within the obese or morbidly obese categories. In physical activity, 44.5% engaged in more than 30 min of exercise daily, whereas 34.3% reported no participation in physical activity. Additionally, 53.1% exercised for three or more hours per week, while 46.9% reported less than three hours of weekly exercise.

**Figure 1:**
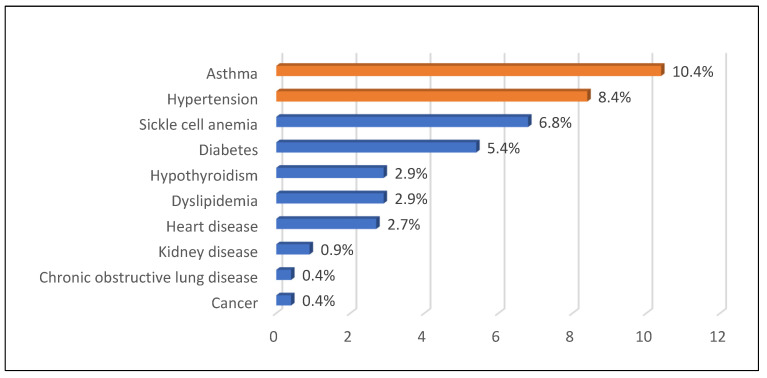
Comorbidities of participants.

With respect to sedentary activities, 68.2% of employees watched television for fewer than three hours per day, and 71.5% slept fewer than eight hours a night. Eating habits showed that 58.5% of individuals consumed fast food one or two times a week, and 22.8% of the study participants never consumed fast food. Comorbidities were relatively low, and asthma (10.4%) and hypertension (8.4%) were the most common comorbidities found (Figure 1).

**Figure 2:**
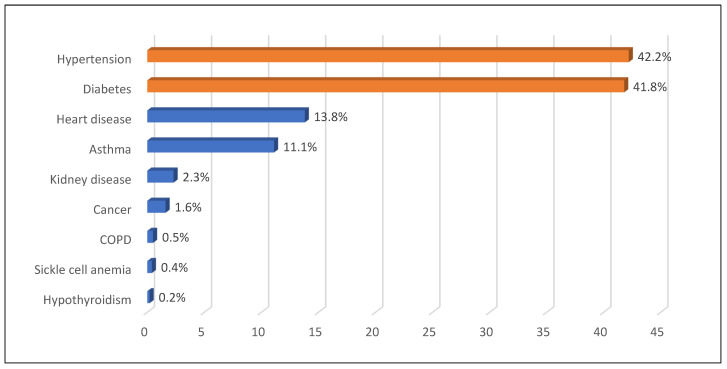
Family history of chronic diseases.

**Figure 3:**
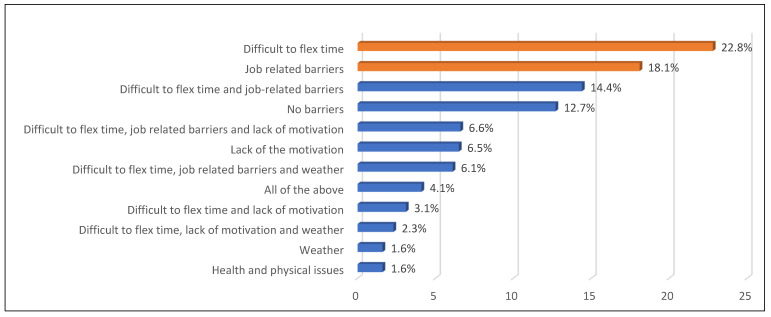
Barriers to exercise.

**Figure 4:**
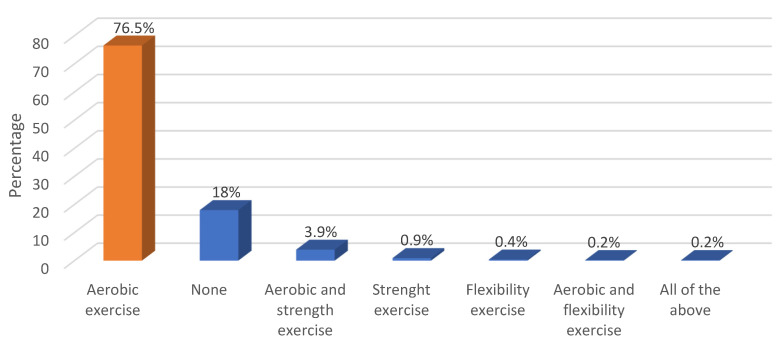
Type of exercise.

Figure 2 illustrates the family history of chronic diseases, revealing that hypertension (42.2%) and diabetes (41.8%) were the most common, while hypothyroidism (0.2%) was the least prevalent. Regarding barriers to exercise (Figure 3), the most commonly reported barriers were difficulty in flex time (22.8%) and job-related constraints (18.1%), while weather conditions and health or physical issues were the least common (both at 1.6%). Aerobics was the most frequently reported form of exercise among employees (76.5%) (Figure 4).

**Figure 5:**
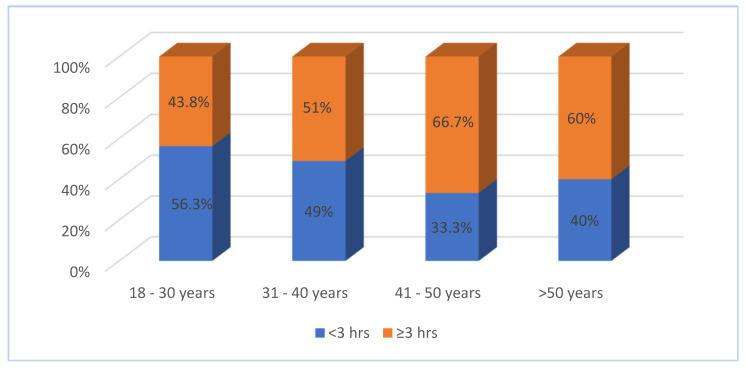
Prevalence of overweight by age group and frequency of exercise per week (N = 188).

**Figure 6:**
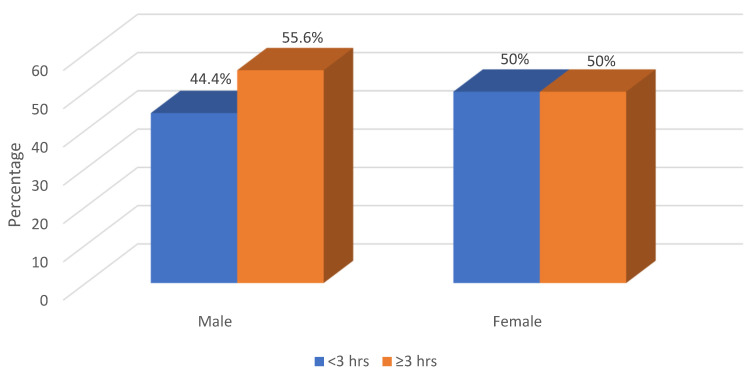
Prevalence of obesity by gender and frequency of exercise per week (N = 104).

**Figure 7:**
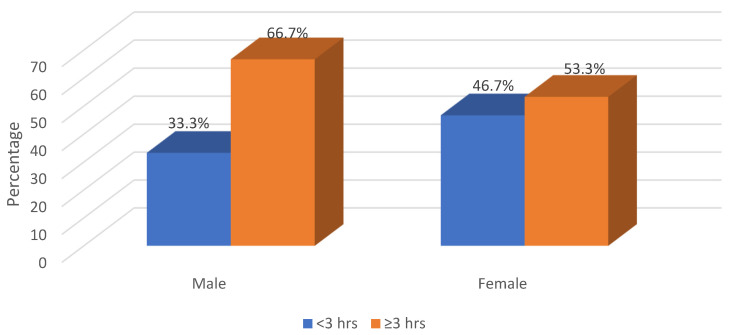
Prevalence of overweight by gender and frequency of exercise per week (N = 188).

With regard to the overweight employees, we determined that those in the 41–50 age group spent more time (≥3 h) doing physical exercise per week (66.7%) than the other age groups, whereas slightly more in the youngest age group (43.8%) spent less time (<3 h) exercising per week (Figure 5). As shown in Figure 6, most of the obese males spend more than 3 h doing exercise per week (55.6%), while no difference in the frequency of exercise per week is seen among obese female employees. As shown in Figure 7, male overweight employees spend more time (≥3 h) doing exercise per week compared to obese females.

When measuring the association between the levels of BMI and the socio-demographic characteristics of employees, we found that those in the older age group (>30 years) are significantly associated with obesity (*p* < 0.001). We also observed that obesity in female employees is statistically significantly higher compared to male employees (*p* < 0.001), while those working in nursing services are significantly more likely to be obese compared to those working in the other departments (*p* = 0.002). Other variables such as nationality (*p* = 0.227), family size (*p* = 0.167), exercise per day (*p* = 0.804), hours of watching TV (*p* = 0.342), hours of sleep per day (*p* = 0.672), and fast food meals per week (*p* = 0.212) did not differ significantly among the levels of BMI (Table 2).

A multivariate regression analysis has been conducted in Table 3 to predict which factors are independently associated with obesity. Multivariate logistic regression allows us to control many variables (e.g., work department, age) simultaneously so that the associations reported are not confounded by other influences. After controlling for other influences, this approach gives us adjusted odds ratios (AORs), which show how much more (or less) likely a person is to be obese based on certain risk factors. Based on the likelihood ratio, we found that the odds of being obese for those employees in the older age group (>30 years) are two times higher compared to those in the younger age group (AOR = 2.404, *p* < 0.001). We also observed that the likelihood ratio of being obese for those employees working in nursing services is almost two times higher compared to those working in the other departments (AOR = 1.999, *p* = 0.003). On the other hand, gender did not have a significant independent effect on obesity when controlling for other variables (AOR = 1.069, *p* = 0.786) (Table 3).

## 5. Discussion

### 5.1. Obesity Among Healthcare Professionals: A Public Health Concern

Obesity among healthcare workers is a significant public health issue affecting workplace productivity, individual health, and healthcare system efficiency. This study confirms a high obesity prevalence among healthcare employees in Al Ahsa, Saudi Arabia, aligning with global trends in the medical profession [15,16]. Specifically, 33.8% of participants were overweight, and 12.9% were obese, emphasizing the need for immediate workplace health policy interventions.

### 5.2. Age as a Key Predictor of Obesity

Healthcare workers over 30 years had twice the obesity risk (AOR = 2.404, *p* < 0.001). Most prior studies point out that such weight gain can be due to metabolic changes through age, or an increase in work-related demands and a concurrent decrease in exercise among healthcare staff [17,18]. Although studies in other countries have often noted a higher prevalence of obesity among males, owing to increased visceral fat accumulation, the prevalence, according to this study, was statistically higher among female workers, with *p* < 0.001 [19]. However, upon multivariate regression analysis, gender was not shown to be an independent predictor for obesity (AOR = 1.069, *p* = 0.786). Thus, confounding factors like sedentary positions and workplace stress are highly active.

Even though health professionals highly understand health matters, work-related limitations lead to unhealthy lifestyle choices such as irregular eating habits, prolonged inactivity, and improper exercise [20]. With AOR = 1.999 and *p* = 0.003, one significant finding of this study was that working in nursing service posed about twice the risk of obesity compared to working in administrative and medical services. This agrees with literature that argues that weight gain among nurses is attributed to shift-based work schedules, extended working days, and higher levels of stress, among other reasons [21]. An astonishing 71.5% of participants slept less than 8 h each night, which was considered alarming. This is an area of concern, as there is already evidence regarding sleep deprivation causing obesity through hormonal dysregulation [22].

In this study, 53.1% of workers reported exercising for at least three hours a week, while 34.3% reported that they did not regularly engage in physical activity. This indicates that sedentary lifestyles remain a problem, especially in healthcare settings. This agrees with the findings published by Okati-Aliabad [23]. Moreoverit was established that workers above 30 years were likely to exercise for at least three hours per week, probably due to increased health awareness and personal drive to reduce the effects of age-related weight gain.

While 31.8% of the respondents reported watching TV over three hours each day, the number of hours was not significantly linked with obesity, this finding was in agreement with Haghjoo et al., [24] (*p* = 0.342). In contrast excessive exposure to screens led to unrelenting snacking and a sedentary lifestyle, which can ultimately result in obesity [24]. 

### 5.3. Patterns of Intake and Fast-Food Consumption in Eating Behavior 

While it has been noted that fast foods are related to a heightened obesity risk due to their high level of calories and low level of nutrient-dense foods, 58.5% of respondents regularly consume it two times per week [25]. Surprisingly, fast food consumption frequency was not significantly correlated with BMI (*p* = 0.212). This indicates that portion control and dietary balance are more serious risk factors for health workers than obesity. On the other hand, individuals with obesity or overweight may adopt healthier behaviors, such as exercising and eating well, due to various factors. Awareness of health risks associated with their weight often motivates them to seek change, while support from friends and family can encourage these efforts. Personal health goals and access to resources like gyms or nutrition programs also play a role. Additionally, engaging in exercise can improve mood and self-esteem, creating a positive feedback loop that reinforces these behaviors. Cultural influences emphasizing wellness may further encourage individuals to prioritize their health, highlighting the complexity of motivations behind their choices.

The fight against obesity among health workers must be undertaken in a multi-dimensional approach with flexible exercises in the workplace, nutrition counseling, and workplace wellness programs as recommended by [26]. Exercises at work during breaks, modification to the workplace in terms of ergonomics, and seminars regarding stress management are some of the recommended interventions that should be prescribed, as job-related constraints (18.1%) and issues relating to time flexibility (22.8%) were the most frequent barriers to exercises. Hospital policies should also provide time for nutritional planning for overnight food consumption, making night workers’ food planning a matter of shift consideration [17].

### 5.4. The Saudi Arabian Context: Cultural, Dietary, and Occupational Influences on Obesity in Healthcare Professionals

Obesity rates among Saudi health professionals are culturally, dietary, and occupationally influenced at very high levels. Traditional Saudi cuisine is dominated by high-calorie, high-carbohydrate foods, including rice-based meals and fried foods, which can be very caloric [27,28]. On top of that, the extremely harsh climate of the country is a limiting factor in outdoor activities during summer periods, which naturally excludes physical exercises for citizens and residents, including healthcare professionals [28]. Moreover, social norms, particularly for females, make it challenging to incorporate physical activity, as cultural barriers to public exercise areas make it difficult for female healthcare workers to frequently engage in physical exercise routines [29]. These, on top of long working hours and shift systems at hospitals, do not favor living a healthy life.

### 5.5. Challenges Facing the Implementation of Health Policy in Saudi Arabia’s Health Sector

Despite the growing awareness of obesity as a public health issue, several challenges impede the implementation of workplace wellness policy in Saudi healthcare institutions. First, while some hospitals have wellness programs, comprehensive, compulsory workplace health programs are still few [30]. Apart from that, health professionals’ poor regard for their health involves poor concern for appropriate mealtimes and physical activities, as they tend to give more emphasis to patient care. In addition, institutional barriers include inadequate availability of structured exercise facilities and of healthy options in the food menus of hospital cafeterias, and inconsistent provision of wellness incentives [2,31]. These factors make the adoption of obesity prevention measures very difficult [32,33]. However, the policies regarding long working hours and shift-work-related stress are not fully covered under occupational health; hence, most healthcare professionals are susceptible to gaining weight due to eating at odd hours and inadequate rest.

### 5.6. Saudi Arabia’s Healthcare Workforce: An International Perspective

Evidence from research indicates that obesity among Saudi healthcare professionals is one of the highest in the world. Compared to Western countries where workplace wellness programs are more organized, Saudi healthcare institutions have fewer system-wide programs that promote obesity prevention initiatives [33]. The policies and programs of hospital-based nutrition education, subsidized gym membership, and work–life balance have also proved effective in the United States and the United Kingdom in lessening obesity among medical workers [34]. For example, Saudi Arabia is ranked among the leaders in obesity rates in the Gulf region, ahead of the UAE and Qatar, both of which have more active policies in terms of the prevention of obesity [35]. Such comparative analyses do show that Saudi healthcare institutions urgently need to adopt and implement structured workplace interventions, like those in Western and neighboring countries, to reduce obesity among health workers.

### 5.7. Implications for Workplace Interventions

The study results provide a very firm basis for workplace health interventions dealing with obesity among health workers, particularly among older employees, females, and nursing staff. It is high time for healthcare institutions to establish organized on-site programs that include physical fitness activities, such as yoga or aerobics after shifts, along with healthy food options at their cafeterias. Besides this, balanced diets can also be promoted and the availability of unwholesome food within health facilities can be restricted by increasing awareness through seminars and workshops.

Stress management programs, which may include mindfulness training, counseling services, and flexible work schedules, are extremely crucial for nursing staff, considering their higher level of occupational stress. Periodic health checkups and awareness programs educate employees on obesity risks and healthier lifestyles.

Obesity-related morbidity leads to absenteeism as well as impaired productivity at workplaces; hence, intervention is required. Saudi hospitals should include prevention of obesity in occupational health policies through fewer hours of undue work, bonus incentives for availing of wellness programs, and appropriate health checkups. The workplace can be further supplemented with ergonomic stationery and relaxing rooms for each employee during their break time. These preventive measures will also create a much healthier workforce without absenteeism issues and with overall quality patient care.

### 5.8. Policy and Workplace Strategies for Obesity Prevention in Healthcare Settings

Prevention of obesity among health workers is a multi-level, systemic issue that requires an integrated approach ranging from national public health programs to workplace wellness initiatives, and down to institutional policies. Health facilities should not make their staff work more than is required and should allow their workers to balance work and family responsibilities to maintain their health. Policies should create an enabling environment where health and well-being are integrated into daily work, for instance, promoting flexible work arrangements and access to mental health resources.

Early intervention programs to reduce obesity risk and routine checkups for health-related problems, as well as compulsory health screening for employees, can be considered by healthcare organizations as part of their organizational policy. Bans on fast foods and cafeteria reform with nutritious meal options are key work site nutrition initiatives. Time set aside in the work schedule, subsidized gym membership, and an available on-site gym can facilitate physical activities at work.

In this regard, leadership is very critical to the creation and normalization of healthier workplace behaviors. Supportive leadership will ensure that such programs—which would encourage employee engagement at the workplace and would include wellness committees, incentive-based health plans, and recognition of personnel for leading healthy lives—will create a culture at healthcare organizations in which decisions are based on health considerations. Any such work policies addressing obesity-related psychosocial and occupational stressors must have programs for stress management, counseling, mindfulness training, and systematic relaxation.

Workplace interventions should be incorporated into national obesity prevention plans to ensure a more holistic and coordinated approach to health. These strategies will provide the necessary base to develop evidence-based, sustainable policies that can be adjusted to various healthcare facilities.

The prevalence of obesity in health workers could be significantly reduced by workplace-driven interventions, systemic policy changes within health facilities, and national health strategies. This approach would increase the quality-of-care delivery, reduce absenteeism rates, and improve work performance overall.

## 6. Limitations

Some strengths of this study include its relatively large sample size, standardized anthropometric measurements, and wide representation of healthcare roles. Limitations include that it is a cross-sectional study that could not allow any type of causal inferences, or probable biased reporting of lifestyle factors. Self-reports are inherently subject to some degree of bias, such as social desirability bias and recall bias, where participants may remember information incorrectly or respond with what they believe is a socially desirable answer. Only one facility population was selected, which reduces the generalization of the results. Future longitudinal studies should replicate the findings of this study in as many healthcare facilities as possible.

## 7. Conclusions

The present study revealed that obesity is highly prevalent among Saudi Arabian healthcare professionals, mainly among older employees and nursing staff. This high prevalence linked to occupational factors strongly calls for including workplace intervention as central to any obesity prevention strategies. Addressing obesity among health professionals is not only a matter of their health but also has become a very important retention factor for an effective and productive health workforce.

Similarly, healthcare centers need to plan and implement some specific programs, including structured exercise initiatives, for employees’ overall health and welfare, stress reduction, and improving diet policies inside hospitals and clinical areas. Easily accessible exercise outlets, nutritional education, and appropriate workplace stress management programs will largely reduce the menace of obesity among the working groups. Moreover, the inclusion of obesity prevention policies will help in adopting healthy institutional practices for better and long-term health conditions. These types of interventions are worthwhile in facilitating staff continuity, performance, and competency in addition to improving personnel health. Hospitals and clinical facilities also need to create institutional policies that support active living in the work environment and healthy nutrition alternatives.

A healthier healthcare workforce will not only improve the health and job satisfaction of individual employees but also lead to higher-quality patient care, reduced absenteeism, increased efficiency, and a healthier, more resilient healthcare system. Obesity among healthcare workers is not just a matter of individual health but a significant determinant for sustaining an effective and high-performing workforce.

Additionally, to induce long-term effects on the health of healthcare professionals, robust prevention measures against obesity must be incorporated into national health initiatives. Future studies should include identification of other job predictors of obesity and evaluation of the effects of workplace wellness programs. Policymakers and health managers will have a viable database to institute more effective and targeted interventions if the long-term effects of such programs are estimated.

Further research is warranted in terms of the evaluation of workplace wellness programs, among other occupational factors influencing obesity among healthcare workers. The long-term impacts of such interventions will prove to be helpful in the drafting of more effective strategies to overcome certain specific challenges facing Saudi Arabian health professionals. A healthier workforce will not only improve the well-being of employees but also enhance the quality of patient care, reduce absenteeism, increase productivity, and, finally, strengthen the healthcare system.

## Figures and Tables

**Table 1:** Socio-demographic characteristics (N = 557).

Study Variables	N (%)
Age group (mean ± SD)	32.7 ± 7.17
• 18–30 years	266 (47.8)
• 31–40 years	216 (38.8)
• 41–50 years	63 (11.3)
• >50 years	12 (2.2)
Gender	
• Male	167 (30.0)
• Female	390 (70.0)
Nationality	
• Saudi	177 (31.8)
• Non-Saudi	380 (68.2)
Number of family members	
• <5	298 (53.5)
• ≥5	259 (46.5)
Department	
• Nursing services	339 (60.9)
• Administration services	85 (15.3)
• Medical services	45 (8.1)
• Support services	88 (15.8)
BMI (mean ± SD)	25.9 ± 5.42
• Underweight	24 (4.3)
• Normal	241 (43.3)
• Overweight	188 (33.8)
• Obese	72 (12.9)
• Morbidly obese	32 (5.7%)
Exercise per day in minutes	
• None	191 (34.3)
• 10–15 min	16 (2.9)
• 20–30 min	102 (18.3)
• >30 min	248 (44.5)
Exercise per week in hours (mean ± SD)	4.73 ± 8.40
• <3 h	261 (46.9)
• ≥3 h	296 (53.1)
Hours of watching TV (mean ± SD)	2.17 ± 1.48
• <3 h	380 (68.2)
• ≥3 h	177 (31.8)
Hours of sleep (mean ± SD)	6.70 ± 1.36
• <8 h	398 (71.5)
• ≥8 h	159 (28.5)
Frequency of eating fast food per week	
• Everyday	20 (3.6)
• One to two times per week	326 (58.5)
• Three to four times per week	75 (13.5)
• Five to six times per week	09 (1.6)
• Never	127 (22.8)

**Table 2:** Association between BMI level and socio-demographic characteristics of employees (N = 557).

Factor	Level of BMI			*p*-Value
	Obese ^(n = 104)^ N (%)	Overweight ^(n = 188)^ N (%)	Others ^(n = 265)^ N (%)	
Age group				
• ≤30 years	32 (30.8)	71 (37.8)	163 (61.5)	<0.001 **
• >30 years	72 (69.2)	117 (62.2)	102 (38.5)	
Gender				
• Male	36 (34.6)	81 (43.1)	50 (18.9)	<0.001 **
• Female	68 (65.4)	107 (56.9)	215 (81.1)	
Nationality				
• Saudi	37 (35.6)	51 (27.1)	89 (33.6)	0.227
• Non-Saudi	67 (64.4)	137 (72.9)	176 (66.4)	
Department				
• Nursing services	49 (47.1)	113 (60.1)	177 (66.8)	0.002 **
• Non-nursing services	55 (52.9)	75 (39.9)	88 (33.2)	
Number of family members				
• <5	47 (45.2)	105 (55.9)	146 (55.1)	0.167
• ≥5	57 (54.8)	83 (44.1)	119 (44.9)	
Exercise per day				
• Yes	67 (64.4)	127 (67.6)	172 (64.9)	0.804
• No	37 (35.6)	61 (32.4)	93 (35.1)	
Hours of watching TV				
• <3 h	65 (62.5)	133 (70.7)	182 (68.7)	0.342
• ≥3 h	39 (37.5)	55 (29.3)	83 (31.3)	
Hours of sleep per day				
• <8 h	72 (69.2)	132 (70.2)	194 (73.2)	0.672
• ≥8 h	32 (30.8)	56 (29.8)	71 (26.8)	
Eating fast food per week				
• Yes	80 (76.9)	153 (81.4)	197 (74.3)	0.212
• No	24 (23.1)	35 (18.6)	68 (25.7)	

The Chi-square (χ^2^) test: association between BMI level and socio-demographic characteristics of employees. **: *p* < 0.001.

**Table 3:** Multivariate regression analysis to predict obesity from selected socio-demographic characteristics of employees (N = 557).

Factor	AOR	95% CI	*p*-Value
Age group			
• ≤30 years	Ref		<0.001 **
• >30 years	2.404	1.509–3.832	
Gender			
• Male	Ref		0.786
• Female	1.069	0.660–1.733	
Department			
• Nursing services	1.999	1.276–3.132	0.003 **
• Non-nursing services	Ref		

**: *p* < 0.001.

## Data Availability

The original contributions presented in the study are included in the article; further inquiries can be directed to the corresponding author.
